# Physico-Chemical Influence of Surface Water Contaminated by Acid Mine Drainage on the Populations of Diatoms in Dams (Iberian Pyrite Belt, SW Spain)

**DOI:** 10.3390/ijerph16224516

**Published:** 2019-11-15

**Authors:** Maria José Rivera, Ana Teresa Luís, José Antonio Grande, Aguasanta Miguel Sarmiento, José Miguel Dávila, Juan Carlos Fortes, Francisco Córdoba, Jesus Diaz-Curiel, María Santisteban

**Affiliations:** 1 Department of Water, Mining and Environment, Scientific and Technological Center of Huelva, University of Huelva, 21004 Huelva, Spain; fefarivera@hotmail.com (M.J.R.); grangil@uhu.es (J.A.G.); aguasanta.miguel@dgeo.uhu.es (A.M.S.); jmdavila@dimme.uhu.es (J.M.D.); jcfortes@uhu.es (J.C.F.); mariasantisteban@gmail.com (M.S.); 2 Sustainable Mining Engineering Research Group, Department of Mining, Mechanic, Energetic and Construction Engineering, Higher Technical School of Engineering, University of Huelva, 21819 Palos de la Frontera, Huelva, Spain; 3 GeoBioTec Research Unit—Department of Geosciences, University of Aveiro, Campus de Santiago, 3810-193 Aveiro, Portugal; 4 Department of Integrated Sciences—Faculty of Experimental Sciences, University of Huelva, 21071 Huelva, Spain; cordobagarciaf@gmail.com; 5 Escuela Técnica Superior de Ingenieros de Minas, Ríos Rosas 21, 28003 Madrid, Spain; j.diazcuriel@upm.es

**Keywords:** acid mine drainage (AMD), benthic diatoms, acidophiles, Pyritic mines, dams, Iberian Pyrite Belt (IPB), Tinto River, Odiel River

## Abstract

Twenty-three water dams located in the Iberian Pyrite Belt were studied during March 2012 (early spring) in order to carry out an environmental assessment based on diatom communities and to define the relationships between these biological communities and the physico-chemical characteristics of the dam surface water. This is the first time that a diatom inventory has been done for dams affected by acid mine drainage (AMD) in the Spanish part of the Iberian Pyrite Belt (IPB). It was found that the pH was the main factor influencing the behaviour of the diatom communities. Then, using a dbRDA approach it was possible to organize the aggrupation of diatoms into four groups in response to the physico-chemical conditions of the ecosystem, especially pH: (1) Maris, Aac, Gos, Cmora (pH 2–3); (2) Andc, San, And, Dpin (pH 3–4.5); (3) Gran, Pleon, Oliv, Lagu, Chan, SilI, SilII, Joya, Gar, Agrio, Camp, Corum (pH 4.5–6); (4) Herr, Diq I, Diq II (pH 6–7). The obtained results confirmed the response of benthic diatom communities to changes in the physico-chemical characteristics of surface water, and helped to understand the role of diatoms as indicators of the degree of AMD contamination in those 23 dams. Special attention was given to those that have an acidophilic or acid-tolerant profile (pH 2–3 and pH 3–4.5) such as *Pinnularia aljustrelica*, *Pinnularia acidophila*, *Pinnularia acoricola* and *Eunotia exigua*, which are the two groups found in the most AMD contaminated dams.

## 1. Introduction

The main problem with sulfide mining is the contamination of water resources caused by acid mine drainage (AMD) processes. AMD occurs when sulfurous minerals are exposed to atmospheric [1], hydrological (oxygen, water) or biological weathering (chemoautotrophic bacteria) and they become oxidized, resulting in sulfuric acid (low pH), dissolved metal ions, elevated sulfate content [2], low alkalinity and high conductivity. The AMD process is widely described in the scientific literature because globally, it is one of the most serious and widespread contamination problems, affecting a large number of water resources in five continents (e.g., [3,4,5]). 

In areas with a semi-arid climate, dam construction is one of the most common solutions to meet the industrial and agricultural water needs of the population. However, the susceptibility of surface water to contamination is much higher than that of groundwater [6]. The problem becomes even more critical when these AMD polluted streams enter water dams, and thus reduce their use. 

The mining of metal sulfide ores is one of the main causes of AMD generation due to the oxidation of the sulphide ore, which creates sulphates and hydrogen ions. This is the case in the Iberian Pyritic Belt (IPB) where sulfide ores have been exploited since Roman times, although there is evidences demonstrating that extractive activities could have begun in 4500 years B.P. [7]. The Spanish part of the IPB comprises 88 mines [8], with most of them generating AMD and an area of more than 4000 ha of waste rock and tailings. The area’s high potential for generating acidity, metals and dissolved sulfates constitutes a huge environmental issue [9].

Thus, from an ecological point of view, AMD defines extreme environments (very low pH values, high metal solubility and the presence of iron colloids) (e.g., [10,11,12]) with a deficiency in carbon and inorganic phosphorus, that are crucial to the efficient functioning of biological communities. Lack of these nutrients increase the impact of stress on organisms, and thus (1) impacted communities experience high metal concentrations and very low pH, which lead to a decrease in the diversity of algal species (i.e., [13,14,15,16]) and (2) communities are limited to tolerant organisms that are capable of surviving in these extreme conditions, and are characterized by a simple ecosystem dominated by acidophilic and acid-tolerant organisms [10,17,18,19,20]. These organisms act as important players, ensuring primary production and interfering in the mobility of dissolved chemical species in the aquatic environment [17,19]. 

Diatoms are one of the main groups of organisms in AMD-affected streams [21]. The Water Framework Directive (WFD), 2000/60/EC [22] introduced a definition of their ecological status for the first time. Knowledge of their ecological characteristics is required to determine reference conditions and pressures, and in order to assess their impact in water bodies. From a monitoring point of view, diatom communities are among the most effective ecological indicators of AMD conditions. Diatoms have been shown to be good indicators of changes in salinity, nutrients and pH, and for this reason, they are routinely used to evaluate different acidification situations [23,24,25] Diatoms are also very sensitive to sudden and negligible changes occurring in the environment [26] and in metal-polluted rivers, they have been shown to respond to perturbations not only at the community level through shifts in dominant taxa [27,28] but also with changes in their diversity [29,30].

In order to carry out an environmental classification based on the diatom communities, especially those that are acidobiontic or acidophilous, 23 AMD affected dams were selected, in the Spanish part of the Iberian Pyrite Belt (IPB). The dams were divided based on the different purposes for storing water: mining, for industrial use and for human supply. In general, this study intended to assess and evaluate the ecological and physico-chemical status of the water dams affected by AMD, and which store water for different purposes. The specific objectives of this work were (1) to describe benthic diatom communities, (2) to assess their relationship with water physico-chemistry specially the pH, and (3) to understand the effectiveness of diatoms as indicators of the degree of AMD contamination, also, several acidic *Pinnularia* species are highlighted as they are among the few organisms able to live in such extreme conditions.

### Study Area

The Iberian Pyrite Belt (IPB) has one of the world’s greatest concentrations of sulfide deposits and extends from Lousal (Portugal) to Aznalcóllar (Spain). The IPB constitutes one of the largest metallogenic regions in the world with approximately 1700 Mt of sulfide reserves [31]. Recently, new reserves have been discovered, proving the existence of important deposits such as in those in Magdalena, with proven reserves of more than 40 Mt [9]. The sulfide ore bodies have been exploited since Roman times and contain pyrite (FeS_2_), sphalerite (ZnS), galena (PbS), chalcopyrite (CuFeS_2_) and arsenopyrite (FeAsS) with other sulfide associated phases of Cd, Sn, Ag, Au, Co, Ni, etc. [32]. Many extensive mining works remain as evidence of sulfide exploitation, along with several million tons of ancient slag [33]. 

Most of these old mining operations were developed in the absence of environmental regulations for discharges. This has resulted in an extensive river network with extraordinary levels of AMD pollution. In many cases, the pollution remains even hundreds of years after the mine closure, with the Tínto and Odiel rivers being the main receptors of these contaminated leachates. Because of this, these two are the most cited rivers in the scientific literature as paradigmatic AMD contaminated rivers.

Most of the 88 inventoried mines from the Spanish part of the IPB [9] generate AMD through mineral-water interaction involving sulfide oxidation (e.g., [34]). Nearly all of the mines were closed before strict environmental guidelines that regulate mining activity were in force. Consequently, no preventive or remediation measures to protect water quality existed. The waters emerging from inside the galleries, as well as the leachates from the waste dumps of the mines are heavily contaminated. Thus, acid mine drainage from these mines merges into a network of tributaries and sub-tributaries [35], until AMD eventually reaches the main two rivers: Tínto and Odiel. 

The semi-arid Mediterranean climate of IPB, with an annual precipitation of around 500–700 mm, generates the problem of water insufficiency. In such a climate, and this hydrological and mining context, water dams have emerged as the best solution to solve the problem of water storage and supply. The Spanish Society of Dams and Reservoirs (SEPREM), officially recognizes 30 dams and reservoirs in the Iberian Pyrite Belt, which are publicly and privately owned, and are used for agricultural, and industrial use, or even for urban supply. AMD reaches the dams and reservoirs via the two most affected rivers, Odiel and Tínto (or, at a larger scale, from the Chanza and Guadiamar rivers,) which have a low pH and a high metal load and sulfates. 

**Figure 1:**
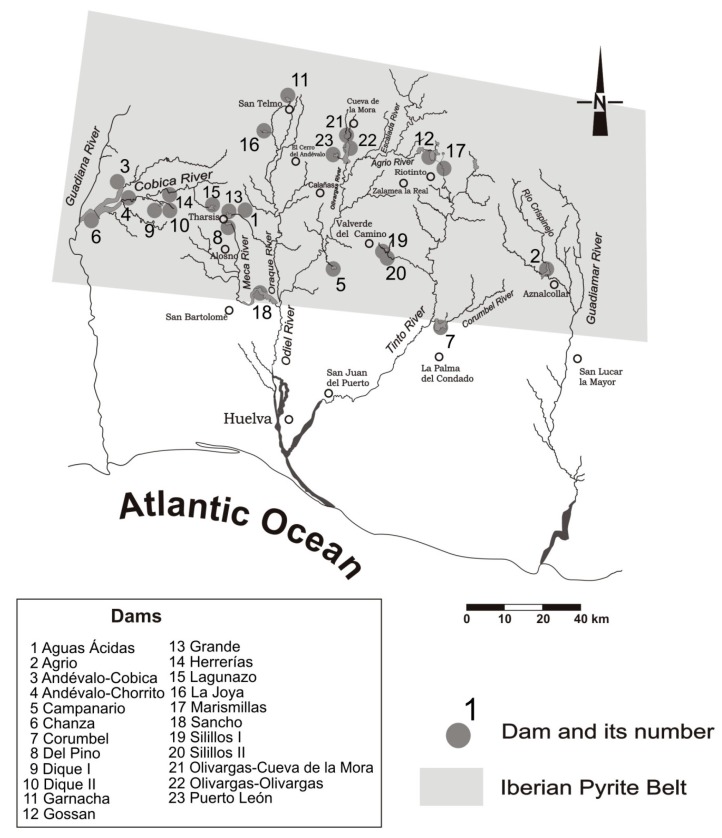
Sampling sites: dams 1–23 located in the Spanish sector of the IPB. (adapted from [37]).

The present work is focused on 23 dams (from the 30 recognized) of the Spanish part of the IPB (Figure 1). They are located in the provinces of Huelva and Seville which encompass the basins of Odiel, Tínto, Chanza and Guadiamar. These rivers cross the Iberian Pyrite Belt from north to south and flow into the Atlantic Ocean into a well-known estuary, named “Ria de Huelva”. Its waters flow with an average pH < 2.5 to the tidal influence zone, which contains a huge dissolved metal load [36].

## 2. Methods

### 2.1. Sampling and Analysis of Water

The sampling period and the selection of the sampling points (Figure 1) was done according to the hydrological context of the study area: diatom sampling was performed at the entrance of each reservoir to investigate the most unfavorable conditions with regard to contamination by AMD, and during spring (March 2012) because this time period provides favorable conditions for the growth and diversity of diatoms.

Determination of the pH, electrical conductivity (EC) and total dissolved solids (TDS) was done in situ using a CrisonMM40^+^ portable multimeter (Crison Instruments S.A., Barcelona, Spain). After this, two water samples were taken and stored in sterilized polyethylene bottles for each sampling point: one for sulfate determination and the other for metals. Nitric acid was added to obtain a drop to pH < 2 in order to prevent metal precipitation during the transport of the sample to the laboratory in a portable refrigerator, at 4 °C.

In the laboratory, the water samples were vacuum-filtered using 0.45-micron cellulose nitrate filters (Sartorius 11406-47-ACN, Göttingen, Germany). Once filtered, the water samples were stored in hermetically sealed polyethylene containers in a refrigerator at 1–4 °C. All the reagents used were of super pure quality (Merck, Darmstadt, Germany). The standard solutions were Merck AA Certificate. Milli-Q water was used in all of the experiments. 

Sulfate concentration was determined through a Macherey-Nagel PF-11 photometer (Macherey-Nagel GmbH & Co. KG, Düren, Germany). Metals and arsenic analysis was done using an atomic absorption spectrophotometer (AAnalyst 800, Perkin-Elmer, Norwalk, USA) equipped with a graphite furnace and an air/acetylene-flame atomizer. The samples were introduced using the Perkin-Elmer AS800 Autosampler (Norwalk, USA). Perkin-Elmer Lumina™ hollow cathode lamps (HDL and LDL) were used as sources of radiation.

### 2.2. Sampling and Analyse of Diatoms

The sampling of diatoms was done according to [37]. The upper sediment diatom samples were collected with a syringe. After collection, one sample per site, was put into light-protected bottles, homogenized and fixed with formaldehyde (4% v) in the field and then transported to the laboratory in refrigerated conditions. A second live sample was collected for qualitative assessment to check the live/dead diatom ratio. Sampling was performed in spring to avoid differences in results due to seasonality, thus drought periods and rainfall events were avoided. In the laboratory, samples went through acid digestion with nitric acid 65% and potassium dichromate (to accelerate the oxidation) for 24 h, followed by several centrifugations at 1500 rpm to wash away the excess acid. Permanent slides with the dried samples were mounted with the high refractive resin Naphrax^®^. Then, diatoms were observed, identified with the highest taxonomic resolution and counted (minimum of 400 valves per sample) using two light microscopes: Olympus CH30 and a Leitz Biomed 20 EB (both in immersion objective 100X, N.A. 1.32) and based on Krammer and Lange-Bertalot [38,39,40,41] floras and other publications (e.g., [18]). OMNIDIA (version 5.3, IRSTEA, Bordeaux, France) was used for information on taxa ecological characteristics. 

### 2.3. Statistical Treatment

#### 2.3.1. Cluster Analysis

For the statistical analysis, cluster analysis techniques were applied to the data related to 16 physico-chemical and two biological variables, the sum of number of diatom species (∑ N° Sp.) and the sum of the relative abundance % of *Pinnularia* species (∑ % Pin) were used to summarize all the data on the species at the 23 sampling sites. 

These two biological variables were used as a summary in the total matrix of 118 diatom species. Cluster analysis has been widely used for the geochemical characterization of AMD media [11,42,43]. Cluster analysis is used to define a series of techniques, basically algorithms, whose objective is to find similar groups of items or variables (sampling points) that are grouped together in clusters. When applied to a set of variables, cluster analysis orders and classifies them in the most homogenous groups possible based on the similarity of the variables themselves.

The program used for this analysis (STATGRAPHICS200 Centurion XVI) operates by applying the Ward method or “second-order central moment”, which is a hierarchical method that calculates the mean of all the variables for each cluster; next it calculates the Euclidean distance between each factor and the mean of its group and then adds the distances from each case. In each step, the clusters that are formed are those that yield the smallest increment in the total sum of the intra-cluster distances. In short, through the application of this technique, the variables studied can be classified into different “categories”. 

First, a linear cluster considering the physico-chemical variables and biological data (the sum of number of diatom species (∑ N° Sp) and sum of the % of relative abundance of *Pinnularia* species (∑ % Pin) as variables was done, and then a second cluster was done considering the sampling sites as variables. A statistical summary was also obtained with STATGRAPHICS200 Centurion XVI.

#### 2.3.2. dbRDA and SIMPER

PRIMER v.6 (Primer 6, Primer-E Ltd., Plymouth, UK) [44] with the add-on PERMANOVA+ [45] was also used. A matrix gathering the diatom responses (% of abundance of 118 taxa, square root transformed, to retain zero values and balance the contribution of rare and dominant species) was used to calculate the Bray-Curtis distance similarity matrix of taxa. On the normalized data of 16 environmental variables (pH, temperature (T), electrical conductivity (EC), total dissolved soils (TDS), metals/metalloids (Al, As, Fe, Cd, Co, Cu, Mn, Ni, Pb, Sb, Zn) and SO_4_^2−^), the Euclidean distance similarity matrix was calculated. A distance-based redundancy analysis (dbRDA) was performed in order to find linear combinations of the predictor variables, which explain the greatest variation in the data in the total matrix of 118 taxa × 23 samples × 16 environmental variables. Distance-based redundancy analysis (dbRDA) is a method for carrying out constrained ordinations on data using non-Euclidean distance measures. dbRDA circumvents this issue using a three-step process: first, a distance matrix is calculated using the distance measure of choice. Next, a principle coordinates analysis (PCoA) is done on the matrix. Finally, the eigenvalues obtained in the PCoA are plugged into an RDA. SIMPER analysis was used to discriminate the species responsible for the largest contribution to the Bray-Curtis dissimilarity in diatom abundance between the samples within each of the four pH groups first discriminated in the dbRDA.

## 3. Results and Discussion

### 3.1. Spatial Distribution of the Environmental Variables 

The statistical summary of 16 physico-chemical and two biological variables, the sum of the number of diatom species (∑ N° Sp.) and the sum of the % of relative abundance of *Pinnularia* species (∑ % Pin) at the 23 sites is shown in Table 1. This collects the basic parametric values of the variables under study. Generally, the statistical summary provides evidence for the presence of a complex water system, shown by the high variability in the physico-chemical and biological parameters (see % variance), which is much higher than would be expected for typical continental waters occurring in similar geo-climatic domains. These abrupt variations point to the existence of severe cases of AMD contamination, which are also highlighted by a very wide pH range (2.21–6.68). As a consequence of this wide pH range (AMD unaffected dams and AMD affected dams) there is a wide range of TDS, EC, SO_4_^2^ and metals (especially, As, Cd, Co, Cu, Fe, Mn, Zn) in dissolution, which is typical of AMD affected systems.

The % of variance is the parameter that provides the most important information, because it indicates what degree of dispersion each variable can take, respecting the central tendency (average). This fact is of crucial importance, especially for the biological variables ∑ % Pin and ∑ N° Sp.: a high value for the first (161.7%) and low value for the second (48.9%) allowed us to define the conditions that control the survival scenario of the species in this geological region (IPB), with *Pinnularia* species having a higher survival capacity in acidic conditions and greater adaptability to different media, depending on the total species (∑ N° Sp) considered in our data matrix. 

The most extreme % variance values are the those for Fe and Cu (> 400%). This is shown in Table 1, indicating the high dispersion values of both, which is not casual considering that there are some sites with pH~7 (max. pH 6.68) and others with very low pH (min. pH 2.21). Fe leaves the waste rock tailings in a reduction environment (pH~3.5; young waters affected by AMD) with high dissolved concentrations in the water. In the presence of oxygen, Fe^2+^ mobilized from iron sulfide oxidizes to Fe^3+^. In general, as long as the pH does not reach 3, the ferric ion precipitates as oxyhydroxisulfate, promoting a decrease in its concentration in solution, while the pH drops due to liberating hydrogen ions [46,47]. According to the previous authors, when the pH is close to 3, the Fe^3+^ concentration increases and acts as a pyrite oxidant without the need for dissolved oxygen, giving the system more Fe^2+^, SO_4_^2−^ and H^+^. 

The lowest % variance values correspond to Pb, Al, temperature and pH. The reasons for this are very different: Pb only reaches 30%, since it is present in very defined paragenesis of the IPB (from galena) and submitted to restricted solubility control conditioned by (pH/Eh) ratios [48]. Al is not part of the primary paragenesis (pyrite) but, on the contrary, its originates on the shales of the enclosing rocks [49]. 

As, Cd, Ni, Co, and Sb did not show significant concentrations in this environment (the range for all variables was 0.001 mg/L–8.6 mg/L). However, it is important to note that the concentration value of As is high: 1.69 mg/L. It is 169 times higher than the limit allowed by [50], which indicates this metalloid has a high toxicity level.

The low % variance for pH (30%) could be misunderstood when compared with the high % variance shown for As, Co (~300%) as well as for Fe and Cu (up to 400%, as explained above). The justification for this discrete % variance is the logarithmic character of pH. 

### 3.2. Biogeochemical Characterization of Physico-Chemical and Biological Parameters from Cluster Analysis

**Figure 2:**
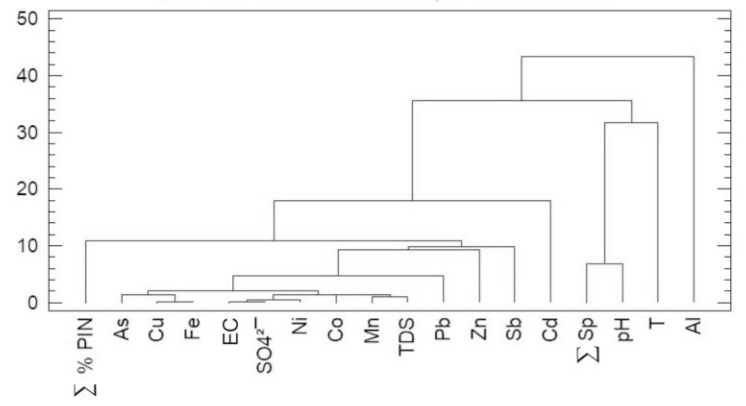
Dendogram resulting from the cluster of physico-chemical and biological variables using the Ward, Euclidean Square Method.

**Figure 3:**
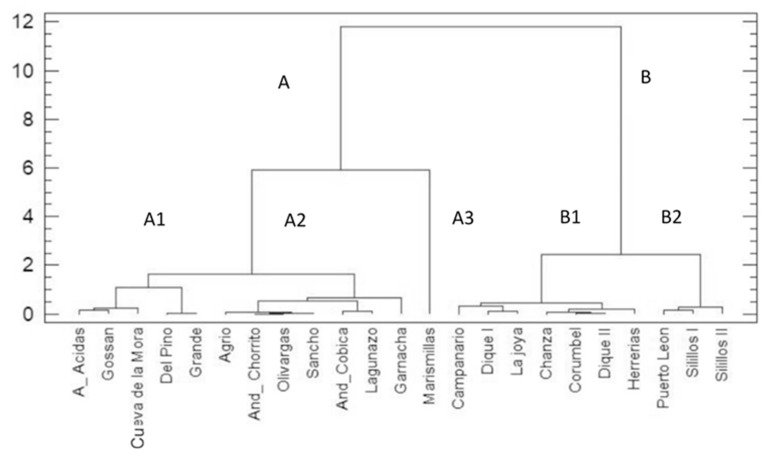
Dendrogram resulting from the cluster of the sampling sites using the Ward, Euclidean.

The dendrograms corresponding to the physico-chemical-biological variables and sampling sites are presented in Figure 2 and Figure 3, respectively. Figure 2, clearly shows the grouping of the variables in two main clusters.

Al is put on its own on the sub-cluster on the far right, and ∑ Sp., pH and temperature (T.) are together in the other sub-cluster on the right. Al is alone because it is not part of the minerals from the primary paragenesis (pyrite), on the contrary, it comes from the shales of the hosting rocks [44]. This fact added to its low solubility ratio compared to the other metals, is what conditions the respective low % variance (44.4%) shown in Table 1. Temperature showed a low % of variance because the range in temperature from different dams is low (maximum of 5 °C), so it does not very much at the sampling sites, and normally it is not an important variable (e.g., [10,17]). ∑ Sp. is closely related to pH since at higher pH there is higher diversity of diatom communities.

The subdivision of the main cluster (on the far left) into two sub-clusters is done according to:

∑ % Pin is opposite to high pH and to high ∑ Sp., since ∑ % Pin is closely related to metals (As from arsenopyrite, and Cu and Fe from pyrite) and to acidic water [10,11,19]). 

TDS is intimately associated with EC in the sub-cluster that shows the highest Pearson’s proximity ratio. This is common in water without dissolved chlorides, which are responsible for the EC variations. In a higher sub-cluster, EC and sulfates were found in close association with AMD typical metals (As, Cu, Fe), as already described by [11]. In this last sub-cluster, the variable TDS appears, which is the sum of the previous variables: EC, sulfates and metals.

High EC is related to high sulfate content (sulfates dissolution caused by pyrite oxidation), which is typical of AMD contaminated waters. 

Mn is not in the sulfur paragenesis but in purple schists on the top of the mineralization [44]. Zn is not bound to Cu as it should be, because its solubility ratio is greater than that of Cu, thus Zn precipitates before, and because of that Cu is not in Zn-sulfate or Zn-oxyhydroxi form, and also Cu is frequently bounded to arsenopyrite [9]. 

Cd, Ni, Co, Sb do not show important or discriminant behavior, as already mentioned in the previous section. Thus, the divisions in this cluster suggest different contamination causality processes in the hydrological environment.

### 3.3. Biogeochemical Characterization of Sampling Sites from Cluster Analysis

The dendogram in Figure 3 shows obvious clustering in two main clusters of samples:

A Cluster A: samples from A_Acidas to Marimillas with pH < 5.68;
B Cluster B: samples from Campanario to Silillos II with pH > 5.70.

Cluster A is divided in 3 sub-clusters:

(A1) A_Acidas, Gossan, Cueva de la Mora, Del Pino, Grande are close together because of their low pH 2.48–4.10, high metal concentration and high ∑ % Pin (32–89%), thus they are very impacted by AMD.

(A2) Agrio, And_Chorrito, Olivargas, Sancho, And_Cobica, Lagunazo, Garnacha have a higher pH range (3.27–5.8) than that of previous group, A1. However, Lagunazo, Agrio and Garnacha have higher pH (5.5–5.9), lower sulfate and lower ∑ % Pin, when compared to other sites from this group. Also due to their higher number of species (∑ Sp.), they are co-located on the rightest side of this sub-cluster.

(A3) Notably, all samples have a very high Pearson proximity ratio, with the exception of Marismillas, which stays at a considerable Euclidean quadratic distance from the rest of the sites of sub-clusters A1 and A2. Marismillas, sticks to a separated sub-cluster, A3. It receives high quantities of dissolved and particulate matter originating from the waste rock tailings of the Rio Tinto Complex mines. Thus, water from this dam comes from the Tinto River and is AMD-affected with acid pH and high sulfate and Fe loads (in this study, ~2.6 g/L of both). Nowadays, this dam is clogged with fine sediments (the TDS here is ~6 g/L coming from the mines, near Nerva village [49,51]. This AMD environment is also subject to spills from Nerva’s Urban Solid Waste Plant (WWTP), which creates different sub-environments, characterized mainly by the availability of organic matter, which does not exist in other AMD contaminated dams. Also, this dam is dominated by *Pinnularia aljustrelica* (∑ % Pin 97%) which could be responsible for its more distant disposition, which is related with the most AMD affected dams (sub-cluster A1). 

Cluster B is divided into two sub-clusters:

(B1) Campanario, Dique I, La Joya, Chanza, Corumbel, Dique II, Herrerias with a pH range of 5.67–6.68 and the highest number of species (∑ Sp.).

(B2) Puerto Leon, Silillos I, Silillos II which is distinguished from the previous group due to its lower pH range of 5.75–5.86 and a medium number of species (∑ Sp.).

### 3.4. dbRDA Analysis

**Figure 4:**
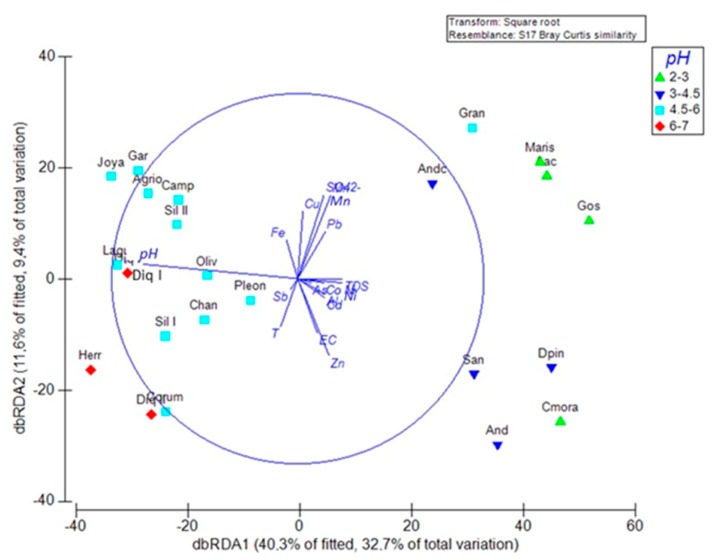
Distance based redundancy analysis (dbRDA) for the diatom resemblance matrix showing the samples’ arrangement and the environmental variables that explain 32.7% + 9.4% of total fitted variation. pH was the main variable that divided treatments into 4 groups: Maris, Aac, Gos, Cmora (pH 2–3); Andc, San, And, Dpin (pH 3–4.5); Gran, Lagu, Pleon, Oliv, Chan, SilI, SilII, Joya, Gar, Agrio, Camp, Corum (pH 4.5–6); Herr, Diq I, Diq II pH 6–7. For the meaning of the dam codes, please see Table 2 where the full name is given.

In this work, the dbRDA (Figure 4) was compared to the statistical results of the cluster analysis for a better understanding of all the processes observed in this scenario. Thus, according to the cluster analysis, pH was identified as the main driving factor of the communities’ and site behavior, so it was selected in dbRDA to define the sampling site groups, according to pH and diatom communities. 

dbRDA has been used previously in similar scenarios [19]. Essentially, dbRDA depicts the overlap of two families of variables involved in the proposed model. The two families of variables could be considered linearly dependent on a first approximation, since part of the information contained in the physico-chemical variables is already included in the information provided the variables, the sampling sites. This is not so linear, since the variables, the sampling sites, in their statistical definition, already included the physico-chemical variables, as well as in the dbRDA. What is new in dbRDA is the matrix of the 118 diatom species (in % of abundance for each species), which was not included in the cluster analysis to avoid the overlap of variables. 

The dbRDA can be considered as a hybridization between a PCA (principal components analysis) and a factorial analysis. This allows the visualization of the physico-chemical variables that show a higher Pearson’s correlation proximity: SO_4_^2−^, Cu, Mn, Pb on one side and EC, TDS, Cd, Co, Al, As, Ni, Zn on the other side and the pH alone, which is the opposite of the previous parameters. pH has a negative significant correlation of −0.830 with the dbRDA coordinate axis 1, separating the two groups on the left of the graph with the highest pH (4.5–7), and, the two groups of samples with the lowest pH (2–4.5) on the right of the graph. Also, SO_4_^2−^ and Mn show an important correlation of 0.45 with dbRDA coordinate axis 2 and are more correlated with sites with low pH: Gran, Maris, Aac, Gos, Andc.

In relation to the grouping of sampling sites, these are defined as four groups: (1) Maris, Aac, Gos, Cmora (pH 2–3); (2) Andc, San, And, Dpin (pH 3–4.5); (3) Lagu, Gran, Pleon, Oliv, Chan, SilI, SilII, Joya, Gar, Agrio, Camp, Corum (pH 4.5–6); and (4) Herr, Diq I, Diq II pH 6–7.

Groups 1 and 2 seem to mix in a certain way, some sites are more related with Cu, Mn, Pb and SO_4_^2−^ (northeast quadrant) and others are more related with EC and the other metals (southeast quadrant). They represent the dams with the highest degree of AMD contamination, which were also grouped together in the cluster of sampling sites. 

dbRDA validates the previous cluster analysis, that is, pH is the most important environmental factor driving the totality of the communities. 

### 3.5. Diatom Community Ecological Preferences: SIMPER Analysis and Graphical Treatment

Analysis of diatom communities shows the species’ contribution to the ecological status of the water dams by considering the frequency and abundance of each taxon, and their relationship to specific physico-chemical conditions. A total of 118 species were found in the 23 water dams, however, many had a low expression (43 species with abundance < 1%). 

**Figure 5:**
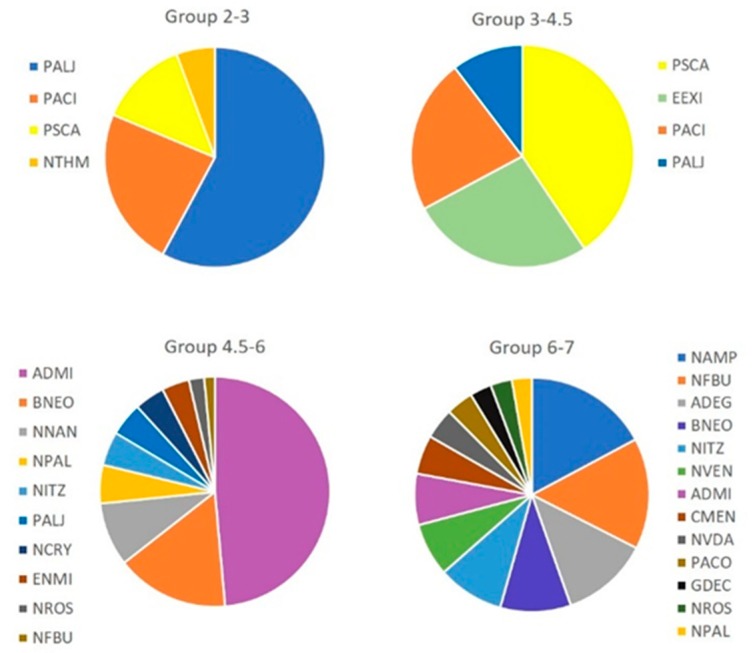
Graphical treatment showing the % of contribution of each species to the observed similarity between samples in each group through SIMPER analysis. Species codes: ADMI- *Achnanthidium minutissimum*, ADEG- *Achnanthidium exiguum*, BNEO- *Brachysira neoexilis*, CMEN- *Cyclotella meneghiniana*, ENMI- *Encyonema minutum*, EEXI- *Eunotia exigua*, PALJ- *Pinnularia aljustrelica*, GDEC- *Geissleria decussis*, NVEN- *Navicula veneta*, NTHM- *Nitzschia thermalis*, NITZ- *Nitzschia sp*., NVDA- *Navicula vandamii*, NFBU- *Nitzschia frustulum* var. *bulnheimiana*, NAMP- *Nitzschia amphibia*, NROS- *Navicula rostellata*, NPAL- *Nitzschia palea*, PACO- *Pinnularia acoricola*, NNAN- *Nitzschia nana*, NCRY- *Navicula cryptocephala*, PACI- *Pinnularia acidophila*, PSCA- *Pinnularia subcapitata*.

From the four groups first in the dbRDA, the dominant diatom species in each group found by SIMPER analysis is presented in Figure 5. This allowed the identification of the most important species, and created similar patterns in samples from each group. At pH < 4.5 (the two first groups), six species known as acid-tolerant were dominant (up to 94% of the total population in these two groups): *Eunotia exigua* (Brebisson ex Kützing) (EEXI) Rabenhorst, *Nitzschia* cf. *thermalis* (Kützing) (NTHM), *Pinnularia acidophila* Hoffman and Krammer (PACI); *Pinnularia acoricola* Hustedt (PACO); *Pinnularia subcapitata* Gregory (PSCA) and *Pinnularia aljustrelica* Luís, Almeida et Ector (PALJ). These species have been found by other authors in similar environments that are characterized by their extreme conditions [10,18,19,20,52]. Thus, species found in these dams fit well with the AMD impact of low pH and high metallic load, which results in the decrease in diatoms diversity, with the spatial variations being more important than the seasonal ones [17].

In dams with a pH of 4.5–6, the total number of species duplicated, increasing the diversity of diatom communities. Acidophilic species do not disappear, however, the neutrophilic species appear to be dominant, for example, *Achnanthidium minutissimum* (Kützing) Czarnecki (ADMI), dominate together with species from the genera *Navicula* (NAVI) and *Nitzschia* (NITZ). 

In dams with a pH of 6–7, the total number of species duplicates is higher compared to the previous group (4.5–6), and this group has the most diverse communities. Alcaliphilic species such as *Nitzschia frustulum* (Kütz.) Grunow var. *bulnheimiana* (Rabh.) Grunow (NFBU), *Nitzschia amphibia* Grunow (NAMP) and *Achnanthidium exiguum* (Grunow) Czarnecki (ADEG) started to appear abundantly. Others, less dominant, such as *Navicula veneta* Kützing (NVEN) and *Achnanthidium minutissimum* (Kützing) Czarnecki (ADMI) also appeared in this group (pH 6–7).

## 4. Conclusions

This was the first time that a diatom inventory was done for AMD impacted dams in the Spanish area of the Iberian Pyrite Belt. The physico-chemical results indicate that the 23 studied dams are subject to sulfates and metallic load from AMD contaminated water courses, although at different levels. The clear response of diatom communities to physico-chemical variations in water was observed through cluster and dbRDA analysis. 

Through cluster analysis, it was noted that three variables: pH, ∑ % Pin and ∑ Sp. mainly conditioned the communities’ and hydrochemical behavior. Marismillas dam was alone in a separated sub-cluster, A3, because of its specific environment (AMD-contaminated waters vs. Nerva’s WWTP inputs), with communities dominated by *Pinnularia aljustrelica* (97%). 

Through dbRDA analysis, the aggrupation of diatoms (with a new variable added: % of abundance of each species and not the sum of number of species in each site (∑ Sp.)) into four pH groups was observed, in response to the intrinsic physico-chemical conditions that govern these ecosystems:

1. Maris, Aac, Gos, Cmora (pH 2–3);
2. Andc, San, And, Dpin (pH 3–4.5);
3. Gran, Pleon, Oliv, Chan, Lagu, SilI, SilII, Joya, Gar, Agrio, Camp, Corum (pH 4.5–6);
4. Herr, Diq I, Diq II (pH 6–7).

The groups of dams were coincident in both cluster and dbRDa analysis, with the exception of Agrio, Garnacha and Lagunazo. These three dams have pH > 4.5 and in dbRDa they were put in the corresponding groups, (pH > 4.5), but in cluster analysis, they were put together with sites of pH < 4.5, due to their higher number of species (∑ Sp.), a variable not included in the dbRDA analysis. 

In summary, each dam presents different degrees of contamination and the existence of a global pattern behavior is not clear. Without rainfall events, it is undoubtedly the pH that controls the metal and sulfate dissolution, which in turn regulates the conductivity values. This absence of a clear global pattern for all of the dams can be interpreted as a consequence of the coexistence of very distinct mineral paragenesis throughout the IPB, as well as the diversity in the size and nature of the watersheds. Other factors, such as the intensity and duration of the mining processes also contribute to the development of individual hydrochemical patterns. Species’ growth and their specific tolerance for distinct pH ranges, especially those tolerant to low pH (e.g., *Pinnularia aljustrelica*, *Pinnularia acidophila*, *Pinnularia acoricola* and *Eunotia exigua*) validates the importance of diatoms as bioindicators of AMD affected dams, contributing to the pH, as well as to the grouping of dams given the diversity of the hydrochemical patterns observed.

## Figures and Tables

**Table 1:** Statistical summary of the 18 variables analyzed in the 23 sampling sites in spring (EC-Electrical Conductivity; TDS-Total Dissolved Soils; ∑ N° Species—Sum of the Number of Species; ∑% Pin—Sum of the total percentage of *Pinnularia* species.

Variables	Average	%Variance	Minimum	Maximum	Range
Al (mg/L)	0.37	44.43	0.07	0.74	0.67
As (mg/L)	0.12	299.66	0.00	1.69	1.68
Cd (mg/L)	0.24	142.60	0.07	1.58	1.52
Co (mg/L)	0.68	279.45	0.01	8.57	8.56
Cu (mg/L)	11.41	426.25	0.03	234.37	234.35
Fe (mg/L)	129.19	413.27	0.13	2559.85	2559.72
Mn (mg/L)	5.75	194.64	0.11	41.63	41.52
Ni (mg/L)	0.18	175.79	0.02	1.23	1.21
Pb (mg/L)	0.34	29.49	0.24	0.61	0.37
Sb (mg/L)	0.02	184.82	0.00	0.13	0.13
Zn (mg/L)	11.59	275.30	0.09	117.78	117.69
SO_4_^2^^−^(mg/L)	474.03	177.17	21.06	3193.63	3172.57
pH	4.79	29.79	2.21	6.68	4.47
T (°C)	16.71	8.94	13.73	19.64	5.91
EC (µs/cm)	1093.81	147.74	157.48	6494.38	6336.90
TDS (mg/L)	8389.7	174.52	100.81	6196.57	6095.76
∑ % Pin	19.93	161.68	0	96.66	96.66
∑ N° Sp.	15.43	48.91	4	27	23

**Table 2:** Main hydrographical characteristics, origin of mine contamination, and water uses of the 23 dams (name and code) under study with locations provided in Figure 1.

Basin	Sub-Basin	River/Stream	Dam/Code	Origin of Mine Contamination	Water Uses
Odiel	MECA	Água Agria	**A_Acidas** (Aac)	Tharsis Group	Industrial-mining
Guadiamar	-	Agrio	**Agrio** (Agrio)	Castillo de las Guardas, Aznalcóllar	Industrial and water supply
Chanza		Malagón	**And_Cobica** (And)	Herrerías and Lagunazo	Urban supply and agricultural
Chanza		Cobica	**And_Chorrito** (Andc)	Herrerías and Lagunazo	Urban supply and agricultural
Odiel	Odiel	Aguas Agrias	**Campanario** (Camp)	Mina Campanario	Recreation and Fishing
Chanza	-	Chanza	**Chanza** (Chan)	Santo Domingo, Vuelta Falsa, el Cura, La Condesa, Sta Ana	Water supply, Fishing, Irrigation
Tinto	-	Corumbel	**Corumbel** (Corum)	Corumbel Group	Water supply and irrigation
Odiel	Meca	Meca	**Del Pino** (Dpin)	Tharsis Group	Industrial
Chanza	-	Chorrito	**Dique I** (Diq I)	Herrerías	Industrial
Chanza	-	Chorrito	**Dique II** (Diq II)	Herrerías	Industrial
Odiel	Oraque		**Garnacha** (Gar)	San Telmo	Industrial-Water supply
Odiel	Odiel		**Gossan** (Gos)	Río Tinto Mining Group	Industrial and Mining
Odiel	Oraque	Água Agria	**Grande** (Gran)	Tharsis Group	Mining
Chanza	-	Chorrito	**Herrerías** (Herr)	Herrerías	Industrial
Chanza	-		**Lagunazo** (Lagu)	Lagunazo	Industrial
Odiel	Oraque	La Joya	**La Joya** (Joya)	La Joya	__
Tinto	-	Tinto	**Marismillas** (Maris)	Río Tinto Mining Group	Industrial
Odiel	Meca	Meca	**Sancho** (San)	Tharsis Group	Industrial-Water supply
Tinto	-	Buitrón	**Silillos I** (Sil I)	Silillos	Water supply
Tinto	-	Buitrón	**Silillos II** (Sil II)	Silillos	Water supply
Odiel	Olivargas	Olivargas	**Cueva de la Mora** (Cmora)	Cueva de la Mora	Industrial, agricultural and water supply
Odiel	Olivargas	Olivargas	**Olivargas** (Oliv)	Cueva de la Mora, Valdelamusa, Sorpresa	Industrial, agricultural and water supply
Odiel	Olivargas	Naranjo	**Puerto León** (Pleon)	Zarza-El Perrunal Group	Industrial-Water supply
